# The inhibitory receptor NKG2A is expressed on T-cells during BKPyVANephropathy

**DOI:** 10.3389/fimmu.2026.1719655

**Published:** 2026-03-25

**Authors:** Karen Bargiel, Christophe Desterke, Manon Dekeyser, Marion Rabant, Lea Dutour, Amelie Grosjean, Arthur Tenenhaus, Amelia Vernochet, Florence Herr, Antoine Durrbach

**Affiliations:** 1 Institut national de la santé et de la recherche médicale (INSERM) 1356, Gustave Roussy Institute, Villejuif, France; 2 Paris-Saclay University, Orsay, France; 3 Institut national de la santé et de la recherche médicale (INSERM)-UMRS1310, Villejuif, France; 4 Department of Nephrology, Centre Hospitalier Régional Universitaire d’Orléans, Orléans, France; 5 Departement d’Anatomopathologie, Hospital Necker, Assistance Publique - Hôpitaux de Paris (AP-HP), Paris, France; 6 Paris University, Paris, France; 7 CentraleSupélec, Gif-sur-Yvette, France; 8 Department of Nephrology and Transplantation, Henri Mondor Hospital, Assistance Publique-Hôpitaux de Paris (AP-HP), Creteil, France

**Keywords:** BKPyVANephropathy, INHIBITORY receptors, kidney transplantation, NKG2A, T-cell exhaustion

## Abstract

**Introduction:**

BKPyV-associated nephropathy (BKPyVAN) is a major complication in kidney transplantation. An exhaustion of BKPyV-specific T-cells but not T-cells against other viruses is observed, suggesting a mechanism specifically mediated by BKPyV-specific CD8^+^ T lymphocytes (BKPyV-LT-CD8).

**Methods:**

We analyzed the proliferation and specific functions in response to stimulation with BKPyV or CEF peptides of T-cells from 41 kidney transplants (23 without BKPyV-DNAemia and 18 with presumed BKPyVAN). We performed comparative transcriptomics on peripheral BKPyV-LT-CD8 and EBV-LT-CD8 cells, and kidney biopsy specimens from patients with BKPyVAN and stable transplants (GSE47199 dataset). The factors identified were validated by immunohistochemistry on graft biopsy specimens.

**Results:**

Levels of the KLRC1 transcript encoding NKG2A were higher in peripheral BKPyV-LT-CD8 than in EBV-LT-CD8 cells. They were also higher in BKPyVAN than in stable kidneys. Immunohistochemistry revealed a higher quantity of NKG2A^+^ cells and of NKG2A^+^ CD8 T-cells in BKPyVAN samples. Furthermore, the NKG2A ligand HLA-E tended to be more abundant in tubular epithelial cells from patients with BKPyVAN than in stable kidneys and located close to NKG2A.

**Conclusion:**

These findings identify NKG2A as a checkpoint inhibitor associated with impaired BKPyV-LT-CD8 function in presumed BKPyVAN. The NKG2A/HLA-E pathway therefore plays a key role in BKPyVAN.

## Graphical Abstract

**Figure:**
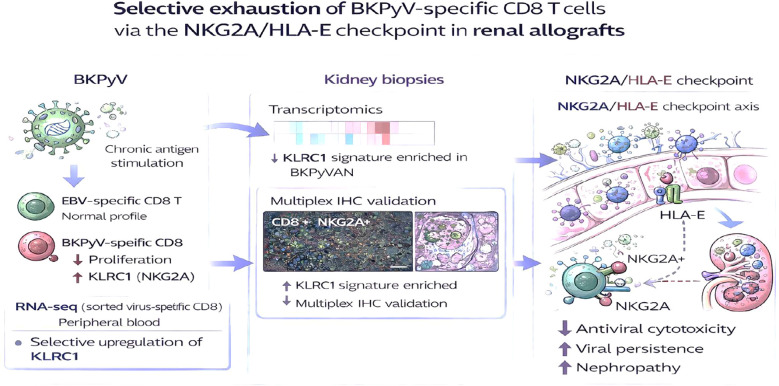

## Introduction

Kidney transplantation (KT) remains the first-choice treatment for patients with end-stage renal disease, providing better survival and quality of life than dialysis (1, 2). However, it necessitates lifelong immunosuppressive therapy, increasing the risks of both cancers and opportunistic infections. Viral infections, in particular, remain a major clinical challenge, despite advances in antiviral therapies.

Polyomaviruses, including BK polyomavirus (BKPyV), are of particular concern in kidney transplant recipients (KTRs) (3). BKPyV is a 5 kb double-stranded DNA virus from the *Polyomaviridae* family. Primary infection is frequent (up to 80% of the population) and usually occurs in early childhood, mostly via oral transmission (4, 5). After a brief viremic phase, the virus enters latency in cells of the urinary tract, including renal epithelial cells and urothelial cells, its natural reservoir. In immunocompetent individuals, transient viral reactivation may lead to low-level BKPyV-DNAuria, but replication is rapidly controlled by the adaptive immune response (3, 6).

By contrast, BKPyV reactivation is frequently observed in immunocompromised individuals, particularly KTRs, affecting up to 40% of them during the first year after transplantation (7–9). The earliest stage is characterized by asymptomatic BKPyV-DNAuria, followed by BKPyV-DNAemia, in up to 25% of patients. Persistent BKPyV-DNAemia (>10^3^ copies/mL over 2 weeks) is associated with the development of BK polyomavirus-nephropathy (BKPyVAN), which affects about 10% of KTRs (3, 6, 10, 11). BKPyVAN is characterized histologically by active viral replication and accumulation in tubular and parietal epithelial cells, leading to viral nuclear inclusions and dystrophy, associated with interstitial inflammation, tubular atrophy, and fibrosis. Ultimately, this disease causes graft loss in up to 50% of affected patients (7–9).

The crucial role of the immune system in controlling BKPyV replication is highlighted by the risk factors associated with BKPyVAN, including T cell-depleting agents, high-dose tacrolimus, and mycophenolate mofetil (7, 12). Early studies suggested a role for HLA mismatches in BKPyVAN, but later observations of disease in HLA-identical donor-recipient pairs and inverse associations between HLA divergence and BKPyVAN risk revealed complex alloimmune and antiviral interactions in this disease. Moreover, polymorphisms in the non-classical HLA-E molecule increase the risk of BKPyVAN, suggesting a possible role for unconventional immune checkpoints (12–15).

Impaired BKPyV-specific T-cell responses independent of global immunosuppression have been described in BKPyVAN (12). These hyporesponsive profiles are characterized by features of T-cell exhaustion, including impaired T-cell proliferation, diminished effector function (cytokine production, cytotoxicity) and an upregulation of inhibitory receptors (IRs) potentially impairing CD8^+^ T cell-mediated viral clearance (12, 15, 16). IRs, such as PD-1, CTLA-4, TIGIT, TIM-3, and LAG-3, modulate T-cell responses via interactions with ligands expressed in the local tissue microenvironment (21–23). Similar exhaustion phenotypes have been reported in chronic infections with other viruses, including HIV, HCV, and HBV, and in cancers (17–20). Specific T-cell hyporesponsiveness has been observed during BKPyV replication, whereas T-cell specific responses against other DNA viruses, such as EBV and CMV, are maintained. This suggests that BKPyV hyporesponsiveness is partly independent of global immunosuppression and is related to BKPyV or graft-associated factors, leading to the exhaustion of specific T-cells in the kidney.

To identify factors associated with the BKPyV exhaustion in renal transplant patients, we studied BKPyV and EBV-specific T cell responses in renal transplant patients. We performed transcriptomic profiling on BKPyV-specific CD8 T-cells, comparing the results with those for other virus-specific T-cells from the same patients to identify the immune regulatory molecules involved in BKPyVAN. KLRC1, encoding the inhibitory receptor NKG2A, was the most abundant transcript in patients with presumptive BKPyVAN. We also found that NKG2A was expressed in renal allograft biopsy specimens from patients with histologically confirmed BKPyVAN, implicating this innate-like checkpoint receptor in BKPyVAN pathogenesis.

## Materials and methods

### Patients

Clinical, biological, and histological data were collected for 41 transplant patients. BKPyV-DNAuria and BKPyV-DNAemia were systematically monitored, monthly, for six months, or as required. Twelve patients displayed no BKPyV replication, 11 had only BKPyV-DNAuria, and 18 had sustained (>3 log/mL) BKPyV-DNAemia. Eight of the patients in this last group had biopsy-proven BKPyVANephropathy. This study (EPUI-BK) was approved by the local research ethics committee (Health Service Research Ethics Committee Ile de France VII ref. PP14-046).

Demographic, clinical, and histological data were recorded for all patients (**Table 1**). BKPyV DNA was quantified by BKPyV-specific real-time PCR according to international guidelines (3). BKPyVANBKPyVAN was diagnosed by kidney biopsy at inclusion, according to international guidelines, with confirmation by immunohistochemistry (SV40 large tumor antigen) (4, 5). Glomerular filtration rate was estimated with the CDK-Epi formula (eGFR). Patients with no BKPyV reactivation or BKPyV-DNAuria were selected randomly from our database of 470 patients.

**Table 1:** Clinical and biological parameters of patients.

Demographic comparison	Without BKPyV-DNAemia	Presumed BKPyVANephropathy	*p^i^*
n	23	18	
Age of patients at transplantation, years	48.8 [27.6-76.3]	67.7 [35.1-74.9]	0.009
Male, *n* (%)	14 (60.8)	13 (72.2)	0.446
DIALYSIS, *n* (%)	20 (86.9)	18 (100)	0.111
Time on dialysis, months	26 [3-113]	29 [12-96]	
Hemodialysis, *n* (%)	18 (78.2)	17 (94.4)	
Peritoneal dialysis, *n* (%)	2 (8.6)	1 (5.6)	
Cause of end-stage renal disease			
Glomerulonephritis, *n* (%)	7 (32)	5 (28)	0.611
Hypertensive or diabetic nephropathy, *n* (%)	5 (24)	7 (38)	
Polycystic kidneys, *n* (%)	4 (18)	2 (10)	
Tubular or interstitial diseases, *n* (%)	1 (6)	2 (10)	
Unknown and other causes, *n* (%)	5 (20)	2 (14)	
TRANSPLANTATION CHARACTERISTICS			
Donation after brain death, *n* (%)	17 (74)	14 (77.8)	0.319
Donation after cardiac death, *n* (%)	2 (8)	2 (11)	
Living donation, *n* (%)	4 (17)	2 (11)	
Highly sensitized patients^ii^, *n* (%)	6 (26)	4 (22)	0.775
First transplant, *n* (%)	21 (91.3)	16 (88.8)	0.89
Pre-existing DSA, *n* (%)	10 (40)	8 (32)	0.95
*De novo* DSA status, *n* (%)	9 (36)	7 (28)	0.987
CMV R+ status, *n* (%)	18 (78)	15 (82)	0.68
Plasma BKPyV viral load (copies/mL) at inclusion	*0*	162351 ± 298901	NA
Time from kidney transplantation to inclusion, months	35 [3.6-177]	9.2 [3-41.8]	0.004
Biopsy-proven BKPyVANephopathy, n	*NA*	8	NA
INDUCTION and MAINTENANCE TREATMENTS			
Induction, *n* (%)	19 (82.6)	18 (100)	0.0625
Thymoglobulin, *n* (%)	14 (77)	11 (61)	0.987
Tacrolimus, *n* (%)	16 (70)	13 (74)	0.852
Belatacept, *n* (%)	3 (13)	5 (27.8)	0.237
Mycophenolate mofetil	23 (100)	18 (100)	, *n* (%)
Mycophenolate mofetil exposure, AUC h.mg/L	42.8 ± 14	52 ± 16.8	ns
CURATIVE TREATMENT FOR REJECTION			
Intravenous steroid boli, *n* (%)	6 (26)	7 (38.8)	0.382
eGFR at month one after transplantation, mL/min/1.73m^2^	40 [32.3-68.8]	51 [31-81]	*0.131*
eGFR at inclusion, mL/min/1.73m^2^	46.0 [27-83]	33.5 [15-63]	*0.123*

### Analysis of BKPyV- or EBV-specific T-cell activation in PBMCs

All T-cell assessments were performed at inclusion, with similar cryopreservation conditions for all blood samples. T-cell proliferation and phenotype were assessed as previously described (6, 7). Cryopreserved PBMCs were thawed, cultured in a 96-well plate after staining with CFSE. They were stimulated with two different BKPyV-specific antigens (PepTivator BKV LT-Ag or VP1, 1μg/mL/peptide, Miltenyi^®^) activating both CD4 and CD8 T cells, or with CEF peptides (peptides pool from cytomegalovirus, Epstein-Barr, and influenza viruses, AXXORA^®^) or staphylococcal-enterotoxin-B – SEB, Sigma-Aldrich^®^) as previously described. T-cell proliferation was assessed by CFSE dilution on CD3^+^ (PerCP, Miltenyi Biotech), CD4^-^(BUV395, BD Biosciences) CD8^+^(APC-Vio700, Miltenyi Biotech) viable cells (BD Horizon™ Fixable Viability Stain 450, BD Biosciences), with a flow cytometer (LSR-Fortessa, BD-Biosciences^®^) and analyzed using FlowJo.v10 software.

### Transcriptomic analysis of peripheral virus-specific CD8 T-cells

Blood samples were obtained at inclusion, with 18 patients presenting sustained BKPyV-DNAemia (>3 log/mL) over the preceding two weeks. PBMCs were isolated from fresh peripheral blood and stained with CFSE (1.25 μM) for 9 min at 37 °C. PBMCs were cultured for 7 days in complete medium (RPMI supplemented with 10% AB serum) under various conditions: negative control (without antigenic stimulation), positive control with 50 µg/mL concanavalin A (Sigma), BKPyV peptides (LT-Ag and VP1 peptides) (PepTivator BKPyV LT-Ag and VP1, Miltenyi Biotec^®^) and EBV peptides (PepTivator EBV Consensus-premium grade, Miltenyi Biotec^®^). On day 7, BKPyV-specific and EBV-specific CD8 T-cells were stained for CD3 (PerCP-Vio700, Miltenyi Biotech), CD8 (APC-Vio700, Miltenyi Biotech), CD4 (BUV395, BD Biosciences) expression and viability(BD Horizon™ Fixable Viability Stain 450, BD Biosciences). Cells were sorted by CFSE dilution on viable CD3^+^CD4^-^CD8^+^ with a sorter (FACS Aria, BD Biosciences) and DIVA software. When possible, 1000 cells per virus were sorted directly into CSS sorting solution (lysis buffer, Takara), centrifuged for 5 seconds, flash-frozen in liquid nitrogen, and stored at -180 °C. For one patient, the number and quality of T cells after sort were insufficient for proper RNA extraction.

RNA was prepared with the SMART-Seq^®^ v4 Ultra^®^ Low-Input RNA Kit for Sequencing (Takara Bio), according to the manufacturer’s instructions. RNA sequencing was performed on a NovaSeq 6000 system (Illumina), generating 40 million reads per sample, by the *Unité de Génomique Fonctionnelle* (Institut Gustave Roussy, IGR).

Quality control, read alignment, and counting were performed on sequencing data by the Bioinformatics Core Facility (BiGR, Institut Gustave Roussy). Reads were cleaned with FastP (8). The GRCh38 human reference genome from Ensembl was indexed by the decoy-aware gentrome method (9), and quantified with Salmon (10). Duplicated transcript sequences were retained during processing, and 100 bootstraps were performed. Counts were aggregated transcripts to genes with TXimport (11), using bootstraps to assess possible additional sequencing bias. Quality report extraction and aggregation were performed with MultiQC (12), the entire pipeline being powered by Snakemake (13).

Bioinformatics analyses were performed with R version 4.1.1. Differential gene expression analysis was performed with the DESeq2 package (14). A volcano plot was generated with the Enhanced Volcano package (15). For exploratory analysis, data were normalized with the Variance Stabilizing Transformation (VST) function of DESeq2. Principal component analysis (PCA) was performed with the factoextra R-package, with visualization of the contribution of the most differentially expressed genes to the first two principal components. A heatmap was generated with the pheatmap R-package (v1.0.12).

### Transcriptomic analysis of graft biopsy specimens (tissue cohort GSE47199)

Normalized microarray data from the GSE47199 dataset (16) were retrieved from the NCBI GEO database (https://www.ncbi.nlm.nih.gov/geo/query/acc.cgi?acc=GSE47199) of data obtained with the Affymetrix Human Gene 1.0 ST Array platform. Probe annotations were based on platform GPL6244 (https://www.ncbi.nlm.nih.gov/geo/query/acc.cgi?acc=GPL6244). The full dataset included 57 samples (40 from blood and 17 from kidney biopsies). We selected 17 kidney biopsies: 3 cases of histologically confirmed BKPyVAN, 3 cases of BKPyV DNAemia with normal biopsies, and 11 control biopsies (normal or with mild interstitial fibrosis and tubular atrophy, IFTA).

Bioinformatics analyses were performed with R software version 4.1.0 (2021–05–18). Supervised differential expression analysis was performed with the limma R-package version 3.48.3 (17). Unsupervised principal component analysis was performed with the “prcomp” R base function. Heatmaps were generated with pheatmap in R (v1.0.12), with Ward.D2 clustering and Euclidean distance metrics. Functional enrichment analyses were performed with Clusterprofiler v4 in R (18) and Gene Ontology – Biological Process (GO-BP) annotations (19). Data were visualized with ggplot2 version 3.3.5 in R (20).

### Immunohistochemistry

Alcohol-formol-acetic acid (AFA)-fixed and paraffin-embedded kidney biopsy specimens were deparaffinized in xylene for 30 min, rehydrated in a graded series of ethanol solutions and antigen retrieval was performed with sodium citrate buffer pH6 in a pressure cooker. Multiplex fluorescence immunohistochemical staining was performed with the following antibodies, conjugated with horseradish peroxidase (HRP) and used consecutively: anti-NKG2A (clone EPR23737-127), anti-CD8 (clone SP16), anti-HLA-E (clone EPR25300-104) (abcam). HRP activity was detected by using fluorophores coupled to tyramides (OPALE, PerkinElmer): Opal 650, Opal 520 and Opal 570 as recommended by the manufacturer. Previously bound primary antibody was stripped before subsequent staining. Multiplex immunofluorescence images were acquired with a Vectra 3 microscope (PerkinElmer). The spectral unmixing of images was performed with inForm software (Akoya Biosciences), followed by quantitative image analysis with QuPath.

### Statistical analysis

Statistical analyses were performed in R version 4.1.0. For transcriptomic data, differential gene expression between groups was assessed with the linear modeling framework of the limma (LInear Models for MicroArray) R-package (version 3.48.3), with empirical Bayes moderation. The resulting *p*-values were adjusted for multiple testing by the Benjamini–Hochberg false discovery rate (FDR) method. Gene expression was compared between sample groups by Fisher’s one-way analysis of variance (ANOVA) followed by Tukey *post hoc* tests for paired comparisons. We considered *p*-values of 0.05 or below (after correction) to be significant for all comparisons.

Non-parametric Mann-Whitney *U* tests (GraphPad Prism10) were used for quantitative comparisons of immunohistochemistry (IHC) results.

## Results

### Impaired specific CD8 T-cell proliferation in BKPyVAN

**Figure 1:**
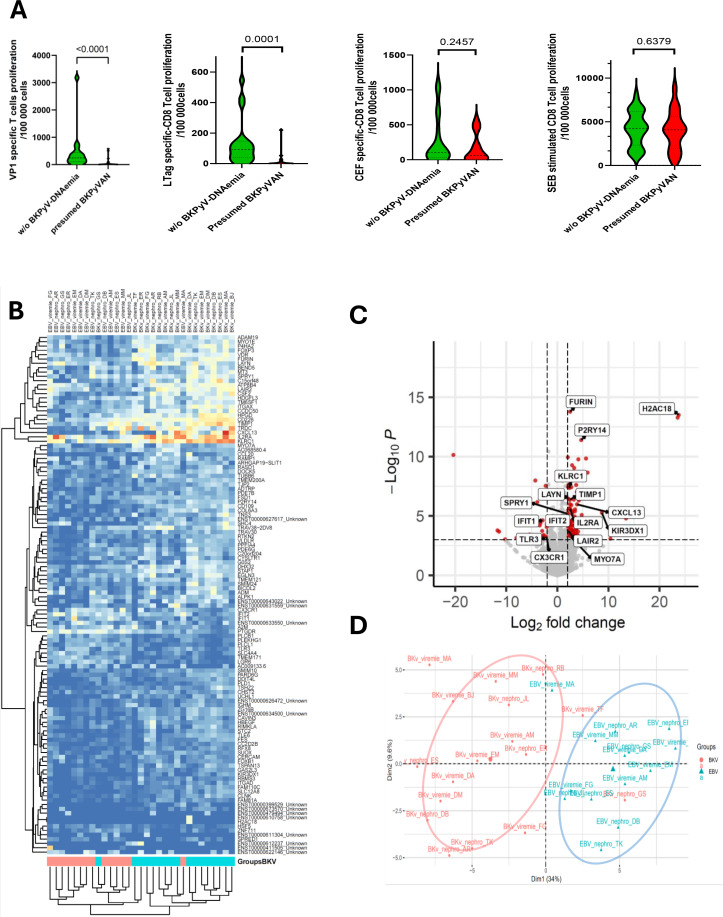
Impairment of BKPyV-specific T-cell function and transcriptomic analysis of differential gene expression between BKPyV-LT-CD8 and EBV-LT-CD8 cells in patients with pBKPyVAN. **(A)** CD8 T-cell proliferation (CFSE low) in patients without BKPyV-DNAemia or with pBKPyVAN, following stimulation with VP1 or LTAg peptides from BKPyV (left panels), or with cytomegalovirus/EBV/influenza peptides (CEF) (middle panel) or SEB (right panel). **(B)** Heatmap highlighting the genes overexpressed in BKPyV-CD8 T-cells. **(C)** Volcano plot presenting differentially expressed genes (log_2_ fold-change >/2/& *p*-adj <0.05). **(D)** Principal component analysis (PCA) plot for the top 120 genes identified in the differential expression analysis.

We evaluated BKPyV-specific cells in 41 patients, 18 patients with presumed BKPyVANBKPyVAN(pBKPyVAN) and 23 transplant recipients without BKPyV-DNAemia (control group). Half the control group had BKPyV-DNAuria (*n* = 11), and the other half had no virus reactivation (*n* = 12). Clinical and biological parameters before and at transplantation are reported in **Table 1**. Only patient age, which was higher in the pBKPyVAN group (*p* = 0.009), and time from transplantation to inclusion in the study, which was higher in the non-BKPyV-DNAemia group (*p* = 0.004), differed between the two groups. Graft function (estimated glomerular filtration rate–eGFR) was similar one-month post-transplantation and was not significantly different at the time of inclusion. BKPyV-specific CD8 T-cell proliferation was lower in the pBKPyVAN group than in controls (*p*<0.0001) (**Figure 1A**). In controls, BKPyV-specific CD8 T-cell counts were similar in individuals with and without BKPyV-DNAuria (data not shown). We then evaluated the counts of CD8 T-cells against other viral peptides (CEF peptides). The specific CD8 T-cell response was similar in the two groups, and no difference in CD8 T-cell proliferation was observed following stimulation with SEB (**Figure 1A**), highlighting the selective impairment of BKPyV-specific CD8 T-cells in transplant recipients with pBKPyVAN.

### Transcriptomic profiling of BKPyV- and EBV-specific CD8 T-cells in patients with pBKPyVAN

We investigated the specific hyporesponsiveness of CD8^+^ T lymphocytes against BKPyV during BKPyVAN, by comparing the transcriptomic profiles of BKPyV-specific CD8 T-cells and EBV-specific CD8 T-cells from the 17 patients with pBKPyVAN. RNA from the EBV-specific T-cells of three patients did not pass quality control and was discarded. By comparing the transcriptomic profiles of BKPyV- and EBV-specific T-cells from the same patients, we identified 120 genes differentially expressed between BKPyV- and EBV-specific CD8 T-cells (absolute fold-change >2, adjusted *p* < 0.05) (**Figure 1B, C**). For 100 of these genes, including *H2AC18, FURIN, P2RY14, KLRC1, TIMP1, CXCL13, IL2RA, KIR3DX1, LAYN, MYO7A*, and *SPRY1*, upregulation was observed in BKPyV-specific CD8 T-cells. Downregulation in BKPyV-specific CD8 T-cells was observed for 20 genes, including that encoding the chemokine receptor CX3CR1, which is highly expressed in T effector cells, the interferon-stimulated genes IFIT1 and IFIT2, and toll-like receptor 3 (TLR3).

Unsupervised principal component analysis on the top 120 differentially expressed genes clearly distinguished between BKPyV-specific and EBV-specific CD8 T-cells (**Figure 1D**). The two major contributors to this distinction were the *CXCL13* transcript, which encodes a chemokine that attracts B cells and T helper to germinal centers, and the *KLRC1 transcript*. Both were overexpressed in BKPyV-specific CD8 T-cells (**Figure 1C** and **S1A**). The *KLRC1* gene encodes NKG2A, an inhibitory checkpoint receptor expressed by natural killer cells and a subset of CD8 T-cells. NKG2A binds to HLA-E, downregulating T-cell activation and effector functions through ITIM-dependent recruitment of the SHP-2 phosphatase.

### *KLRC1* is overexpressed in BKPyVANephropathy

**Figure 2:**
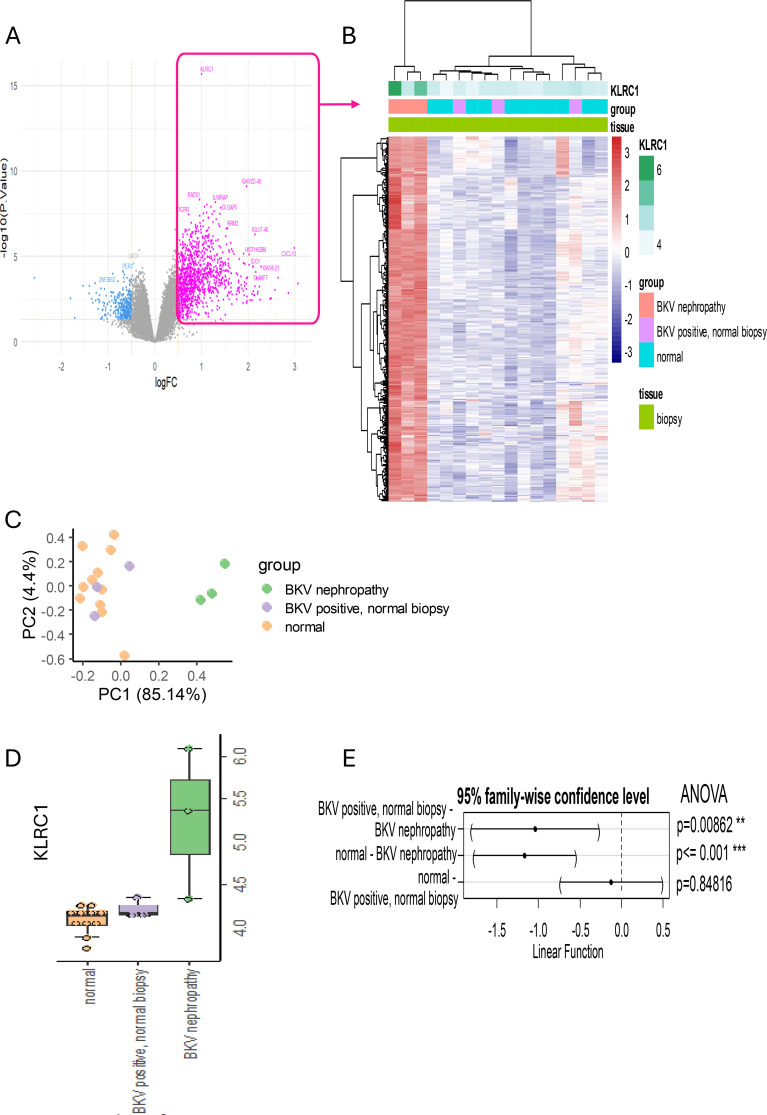
Graphical representation of differential *KLRC1* expression between stable transplanted kidney and BKPyVAN samples (GSE47166). **(A)** Volcano plot highlighting genes significantly dysregulated between stable transplanted kidneys from patients with BKPyV-DNAemia and BKPyVAN. **(B)** Heatmap for genes positively correlated with *KLRC1*, with hierarchical clustering based on transcriptomic profiles for control kidneys (Blue), stable kidney with BKPyV-DNAemia but without nephropathy (Violet), and confirmed BKPyVAN (Pink). *KLRC1* expression was used as a quantitative outcome for the LIMMA algorithm. **(C)** Principal component analysis (PCA) plot separating kidney tissue samples along the PC1 and PC2 axes. **(D)** Boxplot comparing *KLRC1* expression between stable controls, stable controls with BKPyV-DNAemia but without nephropathy, and BKPyVAN. **(E)** Fisher’s one-way ANOVA followed by Tukey *post hoc* tests with 95% confidence intervals, showing intergroup differences in *KLRC1* expression.

We performed transcriptomic analysis on kidney biopsy datasets (GSE47199) to identify the molecules involved in BKPyVAN. *KLRC1* expression was used as a quantitative outcome for the LIMMA algorithm comparing BKPyVAN and other samples (normal stable kidney and stable kidney with BKPyV-DNAemia) (**Figure 2A**, **S1**): the expression of 346 genes was positively correlated with that of *KLRC1*. *KLRC1* was associated with this signature discriminating BKPyVAN from other samples in unsupervised clustering (**Figure 2B**). This *KLRC1* expression signature was upregulated in BKPyVAN (**Figure 2B**). Unsupervised principal component analysis confirmed the separation of BKPyVAN from other samples (**Figure 2C**). Overexpression of the *KLRC1* signature in BKPyVAN samples was confirmed (**Figures 2D, E**), especially in comparison with normal biopsy specimens from patients without BKPyV-DNAemia (*p*<0.0001) and normal biopsy specimens from patients with BKPyV-DNAemia (*p* = 0.008). Functional enrichment analysis on this *KLRC1* expression signature in BKPyVAN highlighted functions relating to immune regulation, such as immune response-cell-surface receptor-signaling pathway, leukocyte-mediated immunity, leukocyte cell-cell adhesion, immune response-activating cell-surface receptor signaling pathway, and lymphocyte proliferation (**Supplementary Figure 1A**). These functionalities shared a large network of molecules suggesting overexpression of several receptors (**Supplementary Figures 1B, C**). In addition, *KLRC1* expression was strongly positively correlated with several immune activation markers, including CD8A and KLRD1, but also the regulatory molecules LILRB1 and LILRB2 (**Supplementary Figure 1B** and **S2B**; r>0.5, *p<*0.001, Spearman’s correlation). These associations suggest a coordinated expression profile linking NKG2A with cytotoxic effector programs, within CD8 T-cells and NK cells, in response to BKPyV infection.

We investigated the immunological pathways associated with NKG2A further, through a gene ontology enrichment analysis based on *KLRC1* expression. Functional enrichment analysis identified biological processes related to immune receptor activity. *KLRC1*-correlated transcripts were enriched in molecular functions including MHC binding, peptide antigen recognition, and inhibitory receptor signaling (LILRB)(p<0.05) (**Supplementary Figures 2A–C**). These data highlight the dual role of NKG2A in antiviral defense and immune regulation in allograft environments.

### NKG2A expression in BKPyV-associated nephropathy

**Figure 3:**
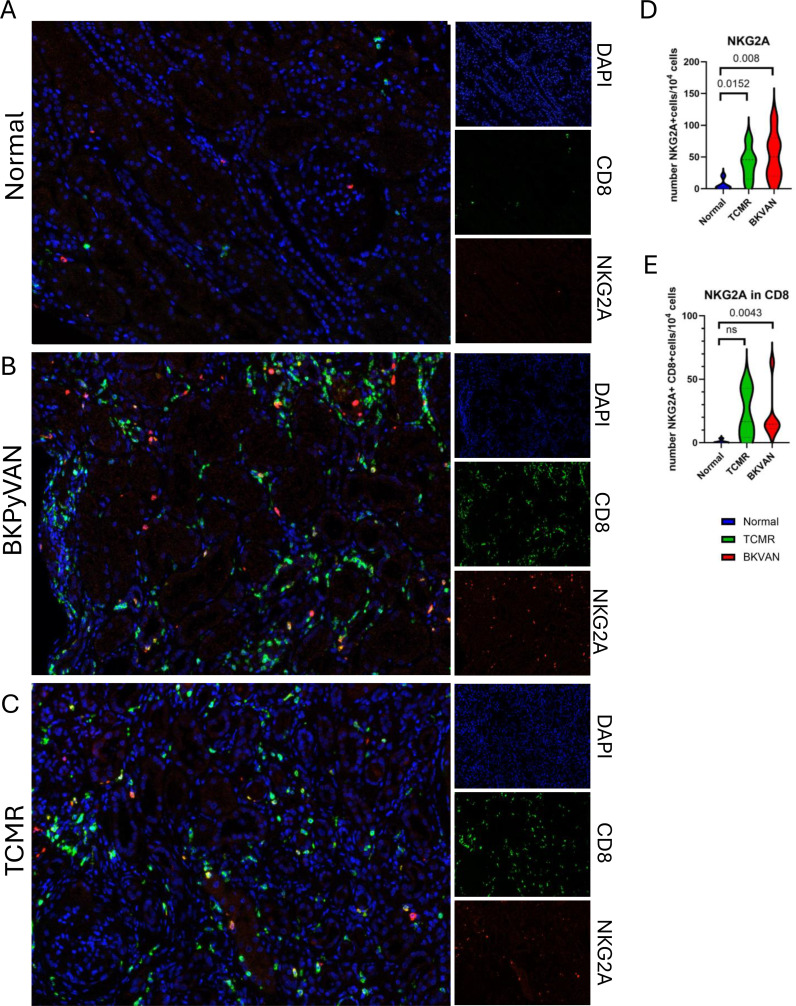
Greater infiltration of NKG2A^+^ immune cells and CD8^+^NKG2A^+^ T-cells in BKPyVAN. Representative immunohistochemistry images **(A–C)** and quantification **(D,E)** of NKG2A^+^ cells and NKG2A^+^ CD8^+^ T-cells in kidney allograft biopsies. **(A–C)** Multiplex immunostaining of NKG2A (red), CD8 **(green)** and DAPI (blue) on normal biopsy specimens **(A)**, BKPyVAN biopsy specimens **(B)** and T cell-mediated rejection biopsies (TCMR) **(C)**, showing enhanced NKG2A^+^ cell and NKG2A^+^ CD8^+^ T-cell infiltration in the tubulointerstitial area of BKPyVAN biopsy specimens than in normal controls. D: Quantification of NKG2A^+^ cell density, showing this density to be significantly higher in BKPyVAN samples (*n* = 6) than in stable kidney (*n* = 5) (*P* < 0.01, Mann–Whitney *U* test). NK2GA^+^ cells were also more frequently observed in TCMR biopsies (*n* = 5) than in stable kidneys. **(E)** Number of NKG2A^+^ CD8^+^ T-cells per 10^4^ cells, revealing significantly greater NKG2A^+^ CD8^+^ cell infiltration in BKPyVAN than in control biopsies (*P* < 0.005, Mann–Whitney *U* test) but not TCMR biopsies. Scale bars: 50 μm.

We assessed NKG2A protein expression in BKPyVAN by evaluating its tissue distribution in renal allograft biopsies. Immunohistochemistry revealed a significantly higher density of NKG2A^+^ cells in the tubulointerstitial compartment of BKPyVAN biopsy specimens than in normal controls (**Figures 3A–D**). NKG2A^+^ mononuclear cells were observed close to tubular epithelial structures and in inflammatory infiltrates. Quantitative analysis confirmed higher NKG2A^+^ cell infiltration rates in BKPyVAN (*P*<0.008, Mann–Whitney *U* test), suggesting a localized immune response potentially regulated by NKG2A (**Figure 3D**). NKG2A levels were also higher in TCMR (T cell-mediated rejection) than in biopsy specimens (**Figure 3C, D**) (*p* = 0.0152). We assessed NKG2A and CD8 coexpression to identify the immune cell subsets responsible for NKG2A expression. Multiplex immunofluorescence analysis showed significantly larger numbers of CD8^+^NKG2A^+^ T-cells in BKPyV^+^ biopsies than in controls (**Figures 3A, B, E**; *p* = 0.0043 Mann–Whitney *U* test). Moreover, 56.25% of CD8+NKG2A+ T-cells were co-expressing PD1, a marker of T cell exhaustion (**Supplementary Figure 3**). Conversely, no enrichment in CD8^+^NKG2A^+^ T-cells was observed in TCMR (**Figures 3A, C, E**, *p*=ns). The high abundance of these cells in the peritubular and interstitial regions in BKPyVAN is consistent with an antigen-driven expansion of inhibitory CD8^+^ T-cells, suggesting that chronic antigenic stimulation may promote NKG2A acquisition in the context of BKPyV infection.

### HLA-E expression in BKPyV-associated nephropathy

**Figure 4:**
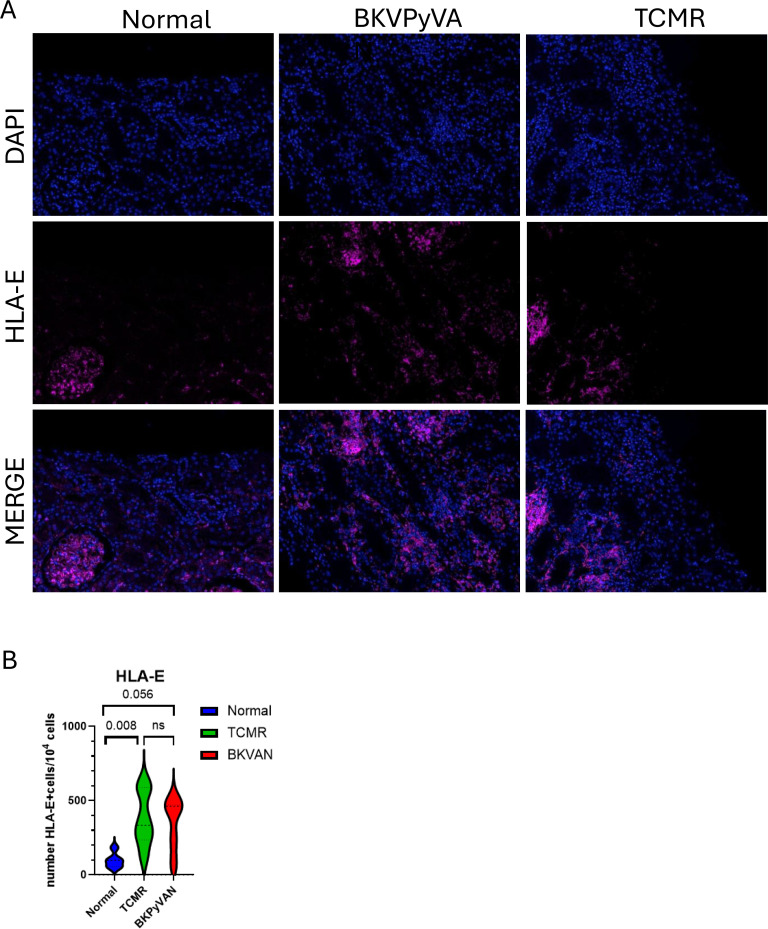
HLA-E distribution in BKPyVAN. Immunofluorescence staining for HLA-E (magenta) and nuclei (DAPI, blue) on kidney biopsy specimens (normal, BKPyVAN, TCMR). Representative image of a BKPyV^+^ biopsy specimen showing enhanced HLA-E expression on tubular epithelial cells **(A)**. Number of HLA-E^+^ cells per 10^4^ cells excluding glomeruli **(B)**. Scale bars: 50 μm.

**Figure 5:**
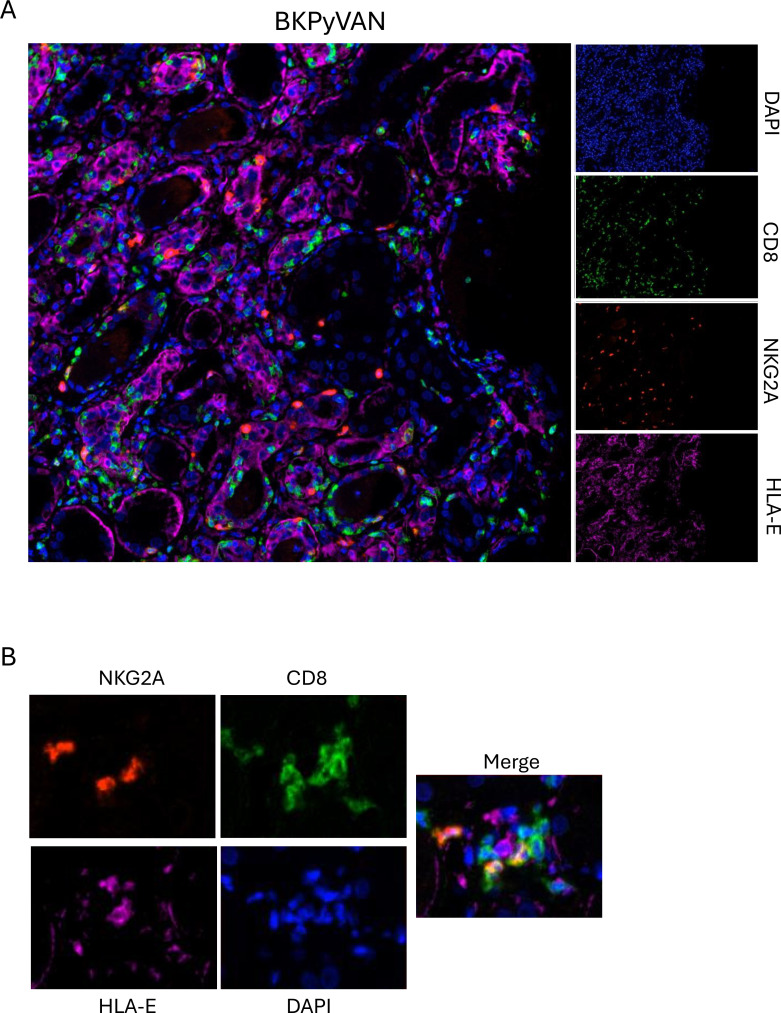
NKG2A^+^ CD8^+^ T-cells interact with HLA-E-expressing cells in BKPyVAN. Multiplex immunostaining of CD8 (green), NKG2A (red), HLA-E (magenta), and nuclei (blue) in BKPyV^+^ tissues, highlighting the interaction of NKG2A^+^ CD8^+^ T-cells with HLA-E-expressing cells in BKPyVAN **(A, B)**. These findings are consistent with activation of the HLA-E/NKG2A axis in the infected allograft.

HLA-E is the ligand of NKG2A. We therefore evaluated HLA-E protein expression *in situ*. In normal kidney, HLA-E was detected in glomeruli and some tubular epithelial cells. In BKPyV^+^ biopsies, HLA-E was markedly upregulated on tubular epithelial cells detected by immunofluorescence imaging (**Figure 4A**). HLA-E levels also increased during TCMR. Quantitative analysis revealed significant tubulo-interstitial expression of HLA-E in TCMR and a tendency for BKPyVAN rather than a stable kidney (*p* = 0.008 and *p* = 0.056, respectively) (**Figure 4B**). In BKPyVAN, higher-resolution multiplex immunofluorescence imaging revealed the presence of CD8^+^NKG2A^+^ T cells close to HLA-E-positive cells in tubule-interstitial infiltrates (**Figures 5A, B**), suggesting functional interactions between these CD8 T-cells and HLA-E-expressing cells.

Collectively, these findings demonstrate that NKG2A is upregulated in BKPyVAN and associated with immunoregulatory transcriptional programs. The concomitant HLA-E expression is consistent with an active inhibitory immune checkpoint, potentially modulating graft inflammation and the outcome of antiviral responses.

## Discussion

We show that the inhibitory receptor NKG2A is upregulated in peripheral BKPyV-specific T-cells and in renal allograft biopsy specimens from patients with BKPyVAN. This upregulation is closely associated with immunoregulatory gene expression programs. NKG2A was co-expressed with CD8 on T-cells infiltrating BKPyV-infected tissues. Moreover, the concomitant overexpression of HLA-E, the canonical ligand of NKG2A (39,40), suggests the establishment of an active inhibitory checkpoint axis in the graft microenvironment.

These findings support the hypothesis that chronic stimulation by BKPyV antigens drives T-cell exhaustion and an upregulation of inhibitory receptors, mirroring phenomena observed in other chronic viral infections (e.g., HIV, HCV, HBV) and cancers, with persistent antigen exposure leading to T-cell dysfunction and unsuccessful pathogen clearance (18–20, 22). Our data indicate that this exhaustion phenotype is not limited to conventional IRs, such as PD-1 or CTLA-4, but also affects innate-like receptors such as NKG2A, potentially impairing viral control despite the presence of virus-specific T-cells (41–43). We found that NKG2A was the principal checkpoint inhibitor distinguishing BKPyV-specific T-cells from other virus-specific T-cells in BKPyVAN. NKG2A is a lectin-like receptor that forms a heterodimer with CD94 to bind the non-classical class-I molecule HLA-E (44–46). Its interaction with its ligand induces inhibitory signaling in NKG2A-positive cells by recruiting intracellular phosphatases that inhibit activation signals in CD8 T and NK cells (47,48). In mouse, chronic polyoma virus infection is correlated with expression of the CD94/NKG2A complex, highlighting its role in chronic infection (49).

The presence of CD8^+^NKG2A^+^ T-cells and transcriptional signatures of immune activation and inhibition in BKPyV^+^ biopsy specimens suggest a dual state of antiviral engagement and regulatory suppression (43). By contrast, no association of NKG2A^+^ CD8^+^ T-cells with TCMR was observed. This functional dichotomy highlights a possible mechanism by which the immune system attempts to balance viral clearance with the allogeneic response in the graft. However, in the context of potent immunosuppression and a high viral load, this balance may be skewed toward tolerance, facilitating persistent infection and progression to nephropathy (50).

The observed upregulation of HLA-E on tubular epithelial cells supports a model in which the local microenvironment actively engages inhibitory pathways. HLA-E expression is upregulated by inflammation and viral stress, enabling epithelial cells to modulate cytotoxic responses and evade immune-mediated injury (51,52). HLA-E expression in epithelial cells is increased by IFN-γ, which may be supplied by activated T-cells in some patients. This checkpoint axis has been implicated in various cancers, including head and neck cancers. In liver, ovarian and colorectal cancers, large numbers of NKG2A^+^ tumor-infiltrating lymphocytes are associated with poor prognosis (53–58). During mouse infection with ectromelia virus, NKG2A knock-out mice show excessive activation of virus-specific CD8+ T cells, suggesting the regulatory function of NKG2A (21). In addition, Zhang and colleagues showed in a mouse model of chronic HCV infection the upregulation of NKG2A on CD8 T cells and/or NK cells and that the use of anti-NKG2A or Qa-1, the mouse HLA-E homolog, blocking antibody improves the HCV clearance (22). The NKG2A-HLA-E axis is, thus, a relevant target for future studies. Furthermore, Rohn et al. have shown that HLA-E polymorphism is associated with BKPyVAN. SNP HLA-E 03*, which is associated with more stable membrane expression of HLA-E, may favor its interaction with NKG2A expressed by BKPyV-specific T-cells, leading to local T-cell exhaustion (59). This hypothesis is supported by our findings showing close contact between HLA-E-expressing epithelial cells and NKG2A^+^ CD8 T-cells. The interaction between HLA-E and NKG2A may mediate immune evasion by BKPyV, via a mechanism reminiscent of those observed in cytomegalovirus and tumor immunology. Collectively, these data suggested that BKPyVAN results from a local interaction in a genetically favorable background between CD8 NKG2A^+^ T-cells and immunosuppression.

Our transcriptomic data also highlights the broader immunological context of NKG2A upregulation, with an enrichment in pathways related to NK cell activation, T-cell receptor signaling, and of the downregulation of immune responses. NKG2A may therefore serve as a molecular integrator of innate and adaptive immunity in the renal allograft, particularly during chronic infections with viruses such as BKPyV. However, based on transcriptomic data, we performed a cell deconvolution to identify cellular signatures associated with BKPyVAN development (data not shown). Our results showed a clear enrichment for CD4 and CD8 memory T cells, macrophages, dendritic cells, B cells, basophiles, but not for NK cells or mast cells. This was confirmed by IHC, which showed that NKp46 cells were less frequent than CD8 T cells (NKp46+ Cells/CD8 T-cells ratio: 11,1 ± 8.7%) in BKPyVAN. These regulatory programs appear to be virus-specific, as BKPyV-specific T-cells display selective exhaustion without a global impairment of antiviral responses.

These findings have important clinical implications. First, they underscore the role of virus-induced immune regulation in BKPyVAN pathogenesis, suggesting that immune exhaustion, rather than simple immunosuppression, may contribute to viral persistence and graft injury. Second, they identify NKG2A as a potential biomarker of T-cell dysfunction in BKPyV infection. Finally, the HLA-E/NKG2A axis may constitute a novel therapeutic target. Monoclonal antibodies blocking NKG2A (e.g., monalizumab) have yielded promising results in oncology and may have applications in infectious diseases based on the restoration of exhausted T-cell function (60,61). In addition, in a chronic mouse HCV model, the impairment of the NKG2A/Qa-1, the mouse HLA-E homolog, axis with blocking antibody targeting NKG2E or Qa-1 improves the HCV clearance, highlighting the important role of this pathway in chronic viral infection. Development of BKPyVAN would be necessary to demonstrate its role in BKPyVAN, which requires HLA-E or its homolog expressed on epithelial cells and NKG2A molecule expressed by immune cells.

This study has several limitations. Although strong associations were found between NKG2A expression and BKPyV-specific immune responses, functional assays are required to confirm the suppressive role of NKG2A in this context. However, they require target cells expressing HLA-E to create the specific interaction between target cells and CD8 T cells expressing NKG2A. Currently, no animal model of BKPyV infection is available to test this hypothesis. In human, longitudinal studies are required to determine whether NKG2A expression predicts progression from asymptomatic viruria to overt nephropathy, and whether it could serve as a biomarker for monitoring disease or guiding immunosuppression. In addition, the analysis of NKG2A expression would also need to integrate HLA-E expression, since HLA-E membrane expression depends on polymorphisms in the recipient population (23). Our work is also limited by the biopsy sample size, which can create some bias based on the focal distribution of the infiltrate and the identification of BKPyV-infected epithelial cells. In addition, the use of paraffin-embedded does not allow us to use multiple staining, and a limited number of markers can be used that pass through the different stages of the experiment.

In conclusion, our study identifies NKG2A as a key immunoregulatory receptor upregulated in BKPyV-associated nephropathy. Its co-expression with HLA-E and cytotoxic markers highlights a virus-specific exhaustion signature that may underline impaired immune control of BKPyV. Targeting of the NKG2A/HLA-E axis may represent a novel strategy for restoring antiviral immunity and protecting renal allografts from virus-driven injury.

## Data Availability

The raw data supporting the conclusions of this article will be made available by the authors, without undue reservation.

## Supplementary material

Supplementary Figure 1 Biological processes for which enrichment was found among genes positively correlated with *KLRC1* expression across kidney transcriptomes (GSE47199): A. Percent contribution of genes to dimension 1 of the principal component analysis. B. Dot plot of Gene Ontology (GO) biological processes identified by enrichment analysis. Processes related to immune activation — such as immune response–regulating receptor signaling, lymphocyte proliferation, and leukocyte-mediated immunity — are prominently represented. Dot size indicates gene count; color gradient reflects statistical significance (adjusted *p*-values). C. Gene-concept network illustrating the associations between selected biological processes and the corresponding genes. Larger gene nodes indicate greater participation across immune pathways. D. Upset plot displaying gene intersections shared between the top-ranking enriched GO terms. Vertical bars indicate the number of genes common to each intersection of pathways, and the matrix below shows which pathways are involved. Enrichment was considered significant at FDR < 0.05.

Supplementary Figure 2 Functional enrichment analysis for genes positively correlated with *KLRC1*. Gene ontology (GO) enrichment plots showing biological processes (A) and molecular functions (B–C) associated with genes positively correlated with *KLRC1*. Enriched terms include “T-cell receptor signaling,” “negative regulation of effector response,” and “cytokine-mediated signaling.” These results further support the dual role of NKG2A in immune activation and inhibition during BKPyVAN. Enrichment was considered significant at FDR < 0.05.

Supplementary Figure 3 Expression of NKGA2, PD-1, PDL-1, CD8 and NKp46 in BKPyVAN. A. Coexpression of C8, PD-1, NKG2A, PDL-1 in biopsies of stable kidney, BKPyVAN or TCR. In BKPyVAN, 56.25% of NKG2A+ CD8 T cells co-expressed PD-1 (n=5). B. Expression of NKG2A, CD8 and NKp46 in BKPyVA. NK cells represent a small proportion of cells, corresponding to 11,1 ± 8.7% of the number of CD8 T cells. 47.1 ± 5.5% were NKG2A positive (n=3).
