# Supplementary figures and images for: The inhibitory receptor NKG2A is expressed on T-cells during BKPyVANephropathy
Source: Front Immunol. 2026 Mar 25;17:1719655. doi: 10.3389/fimmu.2026.1719655 (PMC13056611; doi:10.3389/fimmu.2026.1719655)

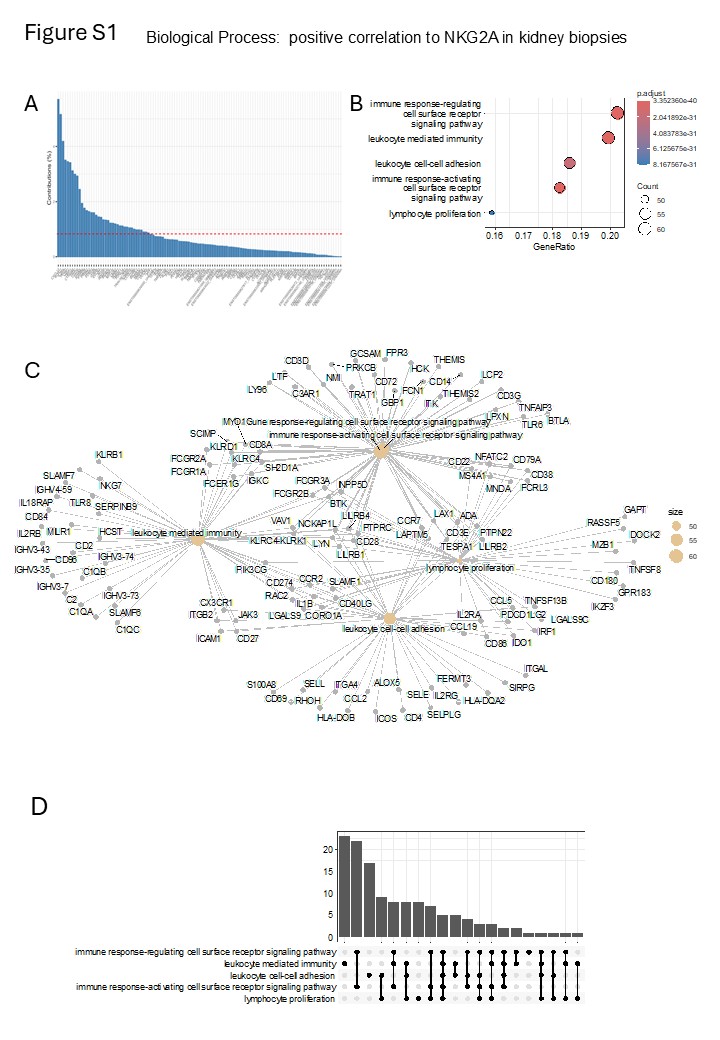

Supplement: Supplementary Figure 1 — Biological processes for which enrichment was found among genes positively correlated with KLRC1 expression across kidney transcriptomes (GSE47199): A. Percent contribution of genes to dimension 1 of the principal component analysis. B. Dot plot of Gene Ontology (GO) biological processes identified by enrichment analysis. Processes related to immune activation — such as immune response–regulating receptor signaling, lymphocyte proliferation, and leukocyte-mediated immunity — are prominently represented. Dot size indicates gene count; color gradient reflects statistical significance (adjusted p-values). C. Gene-concept network illustrating the associations between selected biological processes and the corresponding genes. Larger gene nodes indicate greater participation across immune pathways. D. Upset plot displaying gene intersections shared between the top-ranking enriched GO terms. Vertical bars indicate the number of genes common to each intersection of pathways, and the matrix below shows which pathways are involved. Enrichment was considered significant at FDR < 0.05. [file Image1.jpeg]

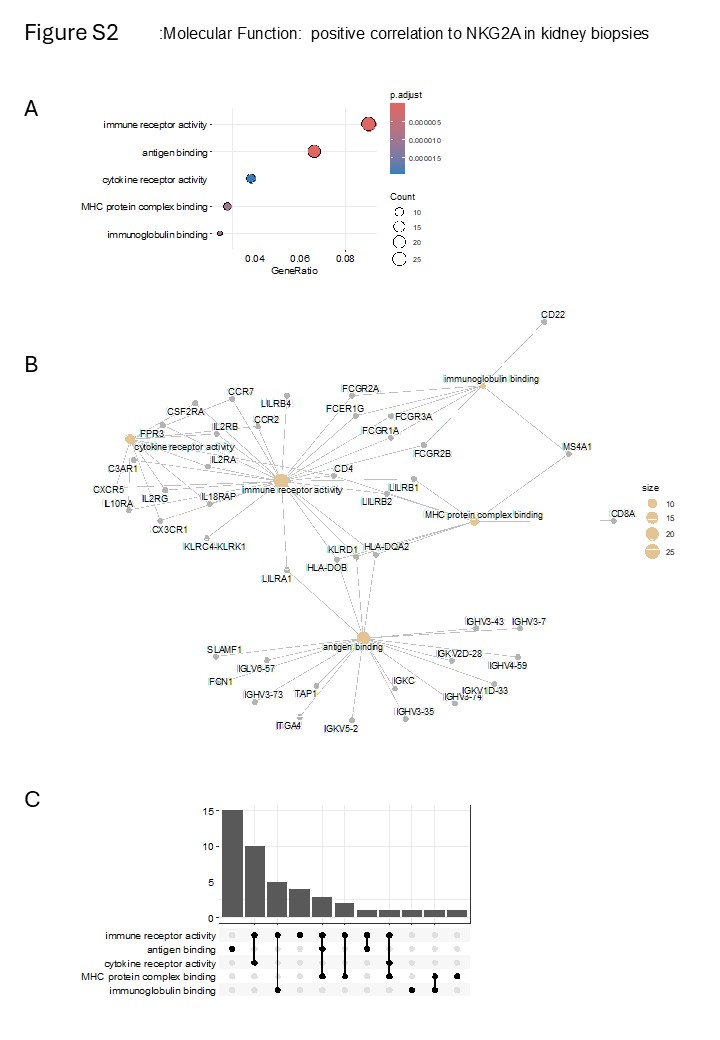

Supplement: Supplementary Figure 2 — Functional enrichment analysis for genes positively correlated with KLRC1. Gene ontology (GO) enrichment plots showing biological processes (A) and molecular functions (B–C) associated with genes positively correlated with KLRC1. Enriched terms include “T-cell receptor signaling,” “negative regulation of effector response,” and “cytokine-mediated signaling.” These results further support the dual role of NKG2A in immune activation and inhibition during BKPyVAN. Enrichment was considered significant at FDR < 0.05. [file Image2.jpeg]

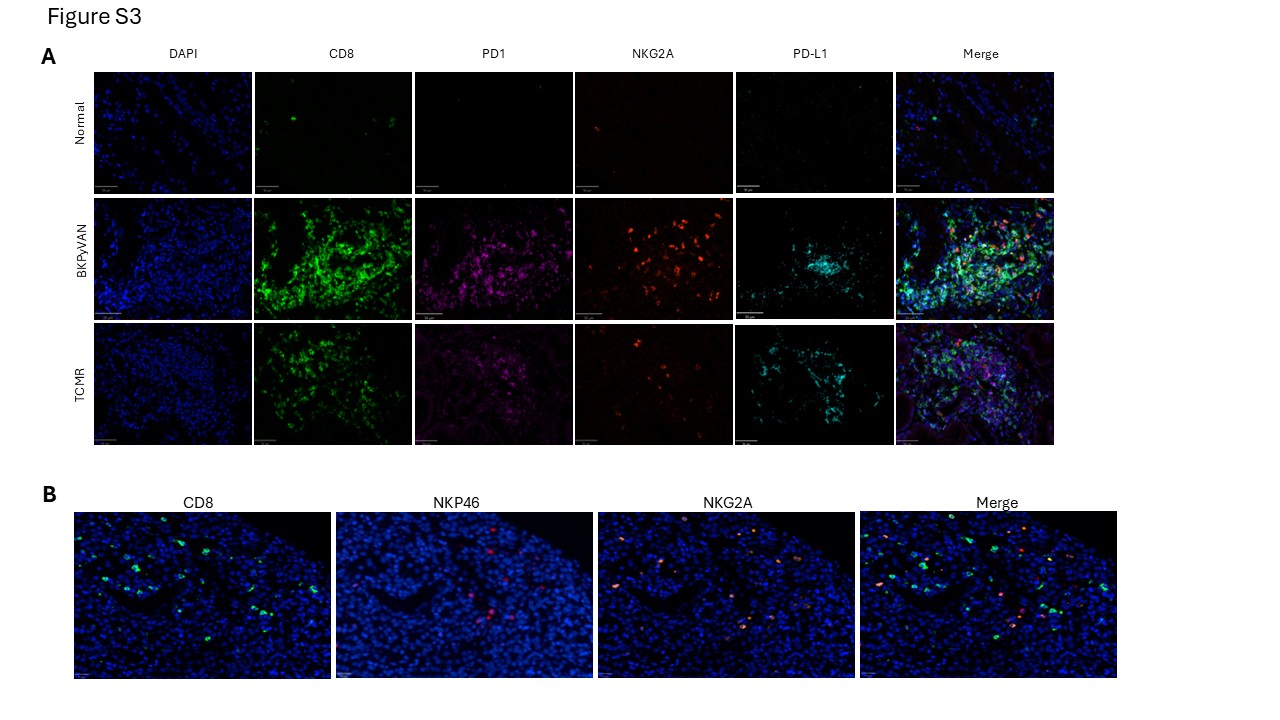

Supplement: Supplementary Figure 3 — Expression of NKGA2, PD-1, PDL-1, CD8 and NKp46 in BKPyVAN. A. Coexpression of C8, PD-1, NKG2A, PDL-1 in biopsies of stable kidney, BKPyVAN or TCR. In BKPyVAN, 56.25% of NKG2A+ CD8 T cells co-expressed PD-1 (n=5). B. Expression of NKG2A, CD8 and NKp46 in BKPyVA. NK cells represent a small proportion of cells, corresponding to 11,1 ± 8.7% of the number of CD8 T cells. 47.1 ± 5.5% were NKG2A positive (n=3). [file Image3.jpeg]
